# (Li_0.4_Co_0.2_Ni_0.2_Cu_0.2_Zn_0.2_)WO_4_: A Novel High-Entropy Wolframite Ceramic with Tailored Microwave Dielectric Properties

**DOI:** 10.3390/ma19071421

**Published:** 2026-04-02

**Authors:** Yutao Sun, Xiong Zhou, Guangshu Feng, Bingli Li, Daode Yang, Dacheng Zhou, Jin Han, Qi Wang, Yong Yang

**Affiliations:** 1 Faculty of Materials Science and Engineering, Kunming University of Science and Technology, Kunming 650093, China; m13099055294@163.com (Y.S.); 19885611826@163.com (X.Z.); fnegguangshu@163.com (G.F.); 18755843117@163.com (B.L.); ydd1187853442@163.com (D.Y.); zhoudacheng@kust.edu.cn (D.Z.); jaakaa@163.com (J.H.); qiwang@kust.edu.cn (Q.W.); 2 Key Laboratory of Advanced Materials of Yunnan Province, Kunming 650093, China

**Keywords:** high-entropy ceramics, microwave dielectric properties, low-temperature co-fired ceramics (LTCC)

## Abstract

(Li_0.4_Co_0.2_Ni_0.2_Cu_0.2_Zn_0.2_)WO_4_ high-entropy ceramics were prepared by a conventional solid-state reaction route. This study thoroughly explores the interrelationships between their crystal structure, bond properties, and microwave dielectric characteristics. X-ray diffraction analysis verified that all specimens crystallized in a single-phase ZnWO_4_-type structure. According to Rietveld refinement of the XRD data, the lattice parameters are affected by the ionic radii of the constituent elements, confirming their dissolution and random distribution at Zn sites. Relative density exhibited a strong dependence on sintering temperature. Bonding analysis highlights the crucial role of the W–O bond in governing the dielectric response of the (Li_0.4_Co_0.2_Ni_0.2_Cu_0.2_Zn_0.2_)WO_4_ (LCNCZW) ceramics. Moreover, sinterability can be improved through optimizing the sintering process. Notably, samples sintered at 850 °C attained suitable dielectric performance, characterized by εᵣ = 11.697 ± 0.204, Q × f = 23,851 ± 0.126 GHz, and τ_f_ = 21.335 ± 0.232 ppm/°C. These results demonstrate that high-entropy design can effectively improve the sinterability and microwave dielectric performance of wolframite-type ceramics, offering a promising strategy for the development of microwave dielectric ceramics for communication devices.

## 1. Introduction

Microwave dielectric ceramics (MWDCs) constitute essential materials for producing critical components such as filters, resonators, and antennas in contemporary communication systems, especially with the expanding implementation of 5G and the Internet of Things (IoT) [1,2,3]. To meet the demands of high-frequency signal transmission and device miniaturization, desirable MWDCs should combine a suitable relative permittivity (balancing signal velocity with the need for component size reduction [4,5]), a high quality factor (Q × f, for minimal dielectric loss), and a near-zero temperature coefficient of resonant frequency (τ_f_) to ensure thermal stability. MWDCs include diverse crystal structures, such as perovskite, corundum, garnet, spinel, olivine, cordierite, zircon, melilite, and tungsten bronze types [6,7,8,9,10,11,12].

Ceramics with wolframite structures have long been a focus of research. Wolframite-type ceramics, including ZnWO_4_, have drawn considerable interest owing to their inherently low permittivity and reasonably high Q × f values. Nevertheless, their characteristics frequently need additional refinement for practical use, especially regarding sintering temperature and thermal stability. For instance, S.D. Ramarao et al. [13] reported the effect of different sintering methods on the crystal structure, lattice vibrational properties, and dielectric performance of ZnWO_4_ ceramics. Zhang et al. [14] reported B-site ion-substituted ZnMo_x_W_1−x_O_4_ (x = 0–0.12) ceramics exhibiting a low permittivity (ε_r_ ≈ 15.10) and a Q × f value of 61,640 GHz, yet their τ_f_ remained a relatively high negative value (τ_f_ ≈ −34 ppm/°C). Furthermore, Tian et al. [15] reported that Pr_2_(Zr_1−x_Ti_x_)_3_(MoO_4_)_9_ ceramics achieved a τ_f_ ranging from −14.4 to −2.6 ppm/°C through ion substitution. An et al. [16] investigated the effects of LiF as a sintering aid on CaWO_4_ ceramics. They reported that LiF addition not only significantly lowered the sintering temperature but also influenced the lattice vibrational characteristics, ultimately leading to improved densification and enhanced microwave dielectric properties. This highlights the critical role of sintering aids in optimizing low-temperature co-fired ceramics (LTCCs).

High-entropy ceramics refer to ceramic systems characterized by a stable single-phase solid solution structure, in which five or more elements in equal or near-equal molar proportions occupy the cationic or anionic sublattices of the crystal. The high-entropy effect, arising from maximized configurational entropy, can stabilize single-phase solid solutions and induce significant lattice distortion and local disorder. These intrinsic structural features are intimately linked to the anharmonic lattice vibrations and bond characteristics that govern microwave dielectric behavior. For instance, severe lattice distortion can enhance phonon scattering, potentially influencing dielectric loss, while the chemical complexity offers a wide ‘compositional space’ to fine-tune the temperature coefficient of resonant frequency (τ_f_). This principle has been successfully demonstrated in recent studies: Zhang et al. [17] showed that the high-entropy ceramic (Nd_0.2_Eu_0.2_Y_0.2_Ho_0.2_Yb_0.2_)VO_4_ exhibited a quality factor more than double that of conventional zircon-structured vanadates, attributed to the entropy-stabilized, highly disordered structure. Similarly, Tian et al. [18] designed the (La_0.2_Nd_0.2_Sm_0.2_Eu_0.2_Gd_0.2_)_2_Zr_3_(MoO_4_)_9_ high-entropy ceramic, which, compared to traditional La_2_Zr_3_(MoO_4_)_9_, demonstrated a reduced sintering temperature and an improved τ_f_, showcasing the potential of high-entropy design to overcome the limitations of conventional microwave ceramics.

Building on this principle, the high-entropy design concept—which incorporates multiple principal cations into a single crystal lattice to form solid solutions—offers a novel pathway for developing advanced materials with unique properties. The increased configurational entropy in high-entropy ceramics can enhance phase stability and potentially influence bonding characteristics and anharmonic lattice vibrations, which govern microwave dielectric behavior.

In this work, we prepared a new wolframite-structured high-entropy ceramic, (Li_0.4_Co_0.2_Ni_0.2_Cu_0.2_Zn_0.2_)WO_4_, and systematically investigated how a multi-cation high-entropy formulation affected the crystal structure, microstructure, and finally the microwave dielectric properties. Given the similar ionic sizes of the included cations (Li^+^, Co^2+^, Ni^2+^, Cu^2+^, Zn^2+^), a single-phase solid solution was anticipated. By clarifying the links between high-entropy structural traits and the resulting εᵣ, Q × f, and τ_f_ parameters, this research intended to supply foundational knowledge for creating high-performance MWDCs via high-entropy engineering.

## 2. Experimental Procedure

The high-entropy ceramic (Li_0.4_Co_0.2_Ni_0.2_Cu_0.2_Zn_0.2_)WO_4_ was produced via a conventional solid-state reaction method. Starting powders of high purity—Li_2_CO_3_ (99%, Aladdin, Shanghai, China), NiCO_3_·2Ni(OH)_2_·XH_2_O hydrate molecular weight 304.12 (99%, Aladdin, Shanghai, China), CoCO_3_·XH_2_O hydrate molecular weight 118.94 (99.5%, Aladdin, Shanghai, China), ZnO (99%, metals basis, powder, <5 μm Aladdin, Shanghai, China), CuO (99%, Aladdin, Shanghai, China), and WO_3_ (99%,metals basis, powder, ≤25 μm, Aladdin, Shanghai, China) were weighed in stoichiometric ratios.

For the preparation of the mixed powders, a planetary ball mill (QM-3SP04, University of Instrument Factory, Nanjing, China) was used with zirconia balls, anhydrous ethanol, and raw materials at a mass ratio of 5:5:1; the milling process was carried out at 300 rpm for 16 h and the result was dried. The resulting powder was compressed into pellets (12 mm diameter, 2 mm height) with 5 wt.% polyvinyl alcohol (PVA) as a binder. Sintering was performed in air using the following protocol: an initial hold at 725 °C for 14 h, followed by heating to temperatures ranging from 800 to 900 °C and maintenance for 4 h. The high-entropy ceramic samples sintered at 800, 825, 850, 875, and 900 °C are hereafter referred to as 800, 825, 850, 875, and 900, respectively.

Crystalline phase identification of both the calcined powder and sintered ceramics was performed using X-ray diffraction (XRD, Bruker D8 Advance, Karlsruhe, Germany) over a 2θ range of 10–80° at a scanning rate of 5° min^−1^. To obtain detailed structural information, Rietveld refinement of the XRD data was carried out using the GSAS-II software package (version latest GSAS-II revision:5782 (svn SVN version 5782)). The refinement procedure included the following parameters: scale factor, background coefficients, lattice parameters, peak profile parameters, atomic coordinates, and isotropic displacement parameters. The quality of the refinement was assessed by the profile factors (R_p_, R_wp_) and the goodness of fit (χ^2^). Unit cell volumes were derived from the refined lattice parameters. Correspondingly, R_wp_ and χ^2^ values have been added to Section 3.

(a, b, c: These denote the lengths of the unit cell along its three crystallographic axes, defining the fundamental dimensions of the crystal lattice. V: This represents the unit cell volume, which is derived from the refined lattice parameters and is critical for understanding structural variations such as densification, doping effects, or phase transitions. Rwp (R-weighted pattern): As one of the most important numerical indicators in Rietveld analysis, Rwp quantitatively assesses the agreement between the observed and calculated diffraction patterns. A lower Rwp value generally signifies a better fit and greater reliability of the structural model). The microstructural morphology and elemental distribution were analyzed with a scanning electron microscope (SEM, Sigma 300, FEI, Hillsboro, OR, USA) equipped with an energy-dispersive spectrometer (EDS). The apparent densities of the sintered pellets were measured using the Archimedes method with deionized water as the immersion medium. For each sintering temperature, three pellets prepared under identical conditions were measured, and the average value with standard deviation is reported. The structural information, chemical composition, and lattice characteristics were analyzed using a Raman spectrometer (Alpha 300, WITec, Ulm, Germany). The microwave dielectric properties were assessed using a vector network analyzer and a temperature-controlled chamber. The relative permittivity (εᵣ) was determined via the Courtney-modified Hakki–Coleman approach, measured at approximately 10 GHz using a vector network analyzer (N5234A PNA-L, Keysight, Santa Rosa, CA, USA), while the quality factor (Q × f) was measured by the standard Hakki–Coleman method. The temperature coefficient of resonant frequency (τf) was evaluated over 25–85 °C based on references [19,20], applying the following expression:

(1) $$\tau_{f}=\frac{f_{85}-f_{25}}{f_{25}\left(T_{85}-T_{25}\right)}$$

where *f*_25_ and *f*_85_ represent the resonant frequencies at 25 °C and 85 °C, respectively.

## 3. Results and Discussion

In this study, a high-entropy ceramic with nominal composition (Li_0.4_Co_0.2_Ni_0.2_Cu_0.2_Zn_0.2_)WO_4_ was designed based on the concept of configurational entropy maximization. According to the Boltzmann entropy formula [21], the ideal configurational entropy on the A-site is calculated as

(2) $${\;S}_{k}=-Nk\sum_{i=1}^{r}\alpha_{i}{\ln\alpha }_{i}=-R\sum_{i=1}^{r}\alpha_{i}{\ln\alpha }_{i}$$

where R is the ideal gas constant, n is the number of different cations on the A-site, and x_i_ is the molar fraction of cation i. For our composition, this yields S_k_ = −R[0.4\ln0.4 + 4 (0.2\ln0.2)] = 1.6R ≥ 1.5R, which exceeds the empirical threshold of 1.5R commonly accepted for high-entropy ceramics. This thermodynamic criterion confirms that the obtained solid solution can be classified as a high-entropy material, providing a foundation for the subsequent investigation of its crystal structure, microstructural evolution, and microwave dielectric properties.

**Figure 1:**
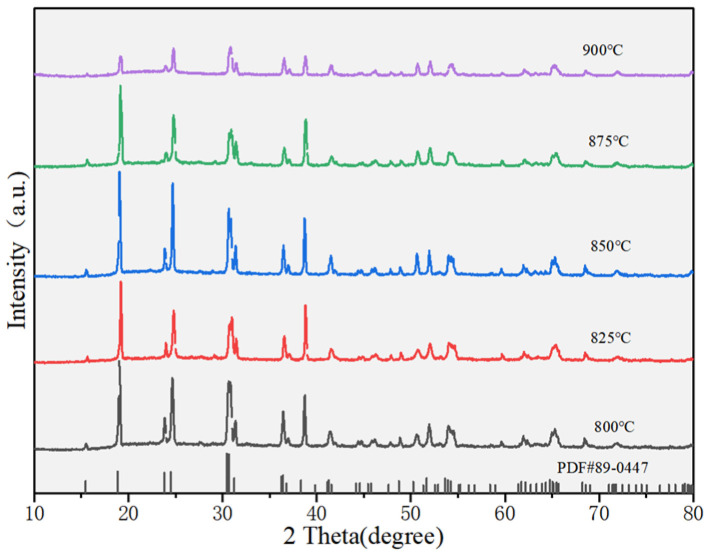
X-ray diffraction pattern of LCNCZW ceramic.

**Figure 2:**
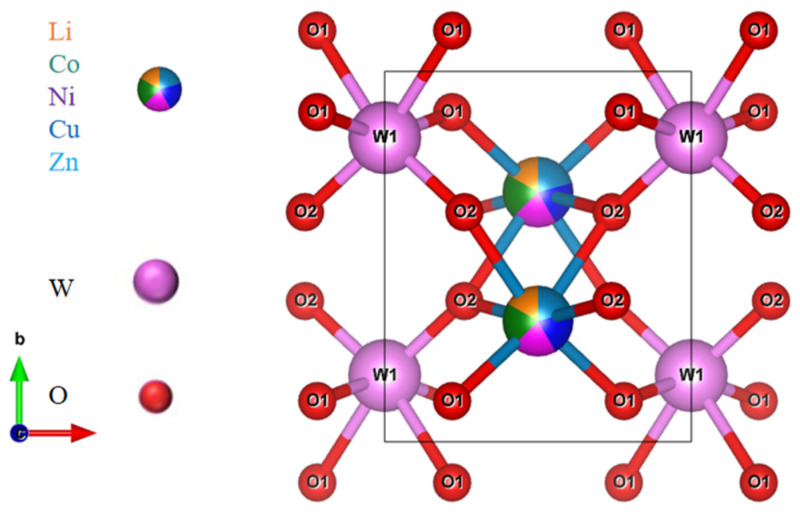
Crystal structure of the LCNCZW ceramic.

**Figure 3:**
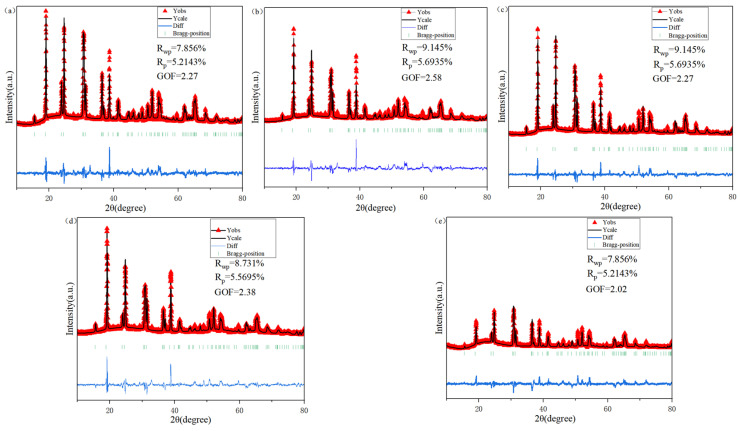
Rietveld refinement plot of the LCNCZW ceramic. (**a**) sinting temperature 800 °C; (**b**) sinting temperature 825 °C; (**c**) sinting temperature 850 °C; (**d**) sinting temperature 875 °C; (**e**) sinting temperature 900 °C.

The X-ray diffraction patterns of (Li_0.4_Co_0.2_Ni_0.2_Cu_0.2_Zn_0.2_)WO_4_ high-entropy ceramics sintered from 800 to 900 °C for 4 h are shown in Figure 1. All samples exhibit a single-phase monoclinic wolframite configuration (PDF #89-0447, space group P2/c) without any detectable secondary phases, confirming the successful formation of a homogeneous solid solution. The incorporation of Li^+^, Co^2+^, Ni^2+^, Cu^2+^, and Zn^2+^ cations into the crystal lattice yields a uniform solid solution—a result aided by their similar ionic radii. A systematic shift of the diffraction peaks to higher 2θ angles is observed, indicating a reduction in interplanar spacing. This effect probably arises from the smaller average ionic radii of Li^+^, Co^2+^, Ni^2+^, and Cu^2+^ compared with Zn^2+^. Accordingly, the unit cell volume decreases, which can be correlated with the contraction of [WO_6_] octahedra induced by the incorporation of smaller cations [22,23]. The crystal architecture of (Li_0.4_Co_0.2_Ni_0.2_Cu_0.2_Zn_0.2_)WO_4_, represented in the inset of Figure 2, includes both Zn and W atoms located at octahedral positions coordinated by oxygen. The Zn site is stochastically occupied by Li^+^, Co^2+^, Ni^2+^, Cu^2+^, and Zn^2+^ ions, while every oxygen ion assumes a three-coordinated geometry. The non-monotonic variation of the unit cell volume with sintering temperature (Table 1) suggests a complex interplay of factors beyond simple thermal expansion. The initial increase in volume up to 825 °C may be associated with the relief of internal stresses accumulated during calcination and the initial stages of grain growth. The subsequent decrease at 850 °C could be attributed to enhanced densification and the elimination of micro-pores, leading to a more tightly packed lattice. At higher temperatures (875–900 °C), the slight volume increase might be linked to subtle changes in oxygen stoichiometry or local cation ordering, which, although not detectable as secondary phases in XRD, can influence lattice parameters. Further investigation using techniques like Raman spectroscopy, which is sensitive to local structural disorder, supports this view, as the FWHM of the main Raman peak remains largely unchanged, indicating that while long-range order is preserved, local fluctuations may occur. Rietveld refinement was performed on all samples to quantitatively evaluate changes in the lattice parameters and crystal structure. The excellent agreement between the observed and calculated patterns for all sintering temperatures is demonstrated in Figure 3, and the corresponding refinement parameters, including the GOF, are listed in Table 1 [24]. 

**Figure 4:**
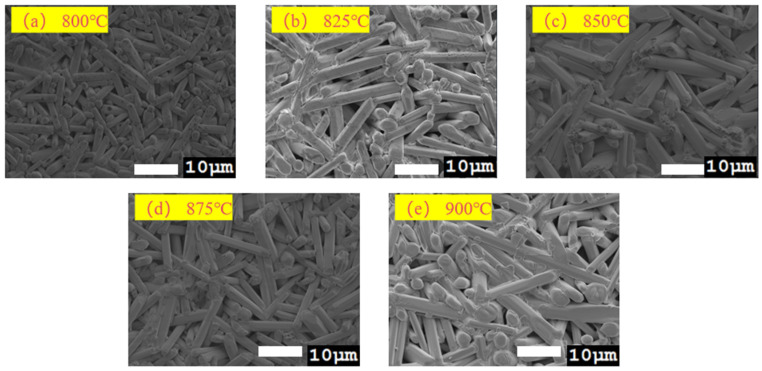
SEM images of the LCNCZW ceramic sintered at 800–900 °C.

**Figure 5:**
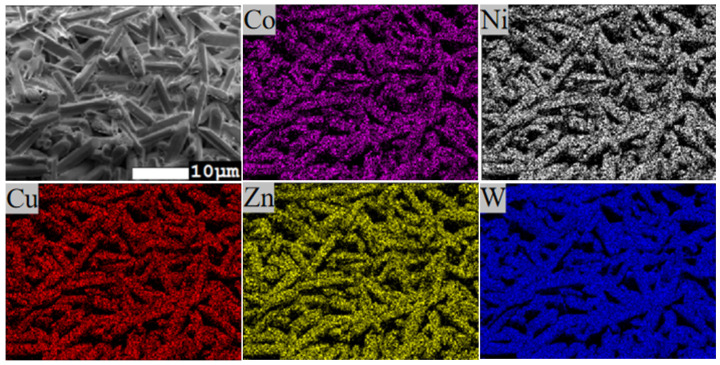
EDS spectrum of the LCNCZW ceramic sintered at 850 °C.

SEM observations of the surface morphology (Figure 4) show that as the sintering temperature increases from 800 °C to 850 °C, the grains gradually grow and densification improves. At 850 °C, the sample surface exhibits the largest grain size and the densest microstructure, consistent with the maximum bulk density achieved at this temperature. When the temperature is further increased to 900 °C, the surface remains dense, but the bulk density shows a slight decrease. This phenomenon is typically associated with over-sintering, which may lead to the formation of a small number of pores at grain boundaries. Furthermore, energy-dispersive spectroscopy (EDS) analysis was conducted on the ceramic. As shown in Figure 4, the constituent elements—Co, Ni, Cu, Zn, and W—are uniformly distributed throughout the (Li_0.4_Co_0.2_Ni_0.2_Cu_0.2_Zn_0.2_)WO_4_ bulk ceramic, with no evidence of elemental segregation observed. The dark regions observed in the SEM image (Figure 5) are attributed to surface topography or residual porosity, rather than compositional segregation, as the corresponding EDS maps show a uniform distribution of all elements across the entire area, including these dark regions.

**Figure 6:**
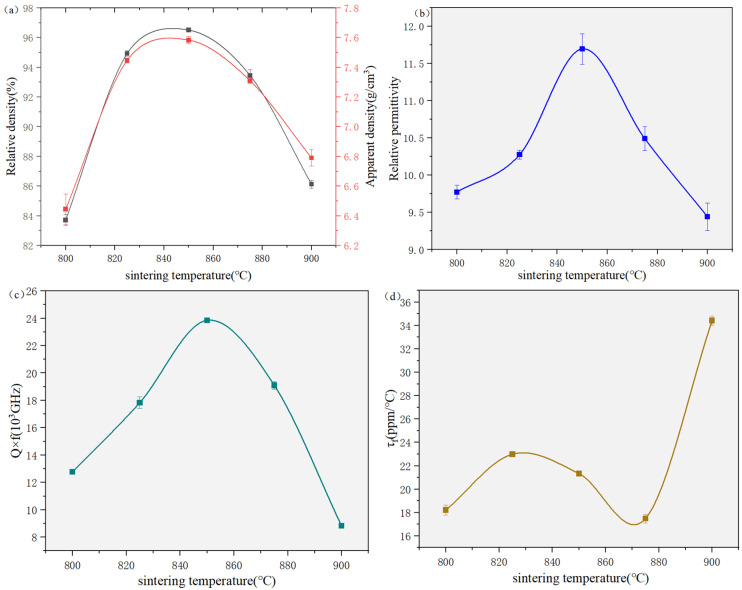
Properties of the LCNCZW ceramic as a function of sintering temperature: (**a**) apparent and relative density, (**b**) relative permittivity, (**c**) Q × f value, and (**d**) τ_f_ value.

The apparent density of the LCNCZW ceramic as a function of sintering temperature is illustrated in Figure 6a. The apparent density increases from 6.553 g/cm^3^ to 7.580 g/cm^3^ as the sintering temperature rises to 850 °C, which could be attributed to the progressive elimination of pores and enhanced densification. When the temperature was further increased, over-sintering leads to abnormal grain growth and the re-formation of pores, resulting in a gradual decrease in apparent density. The dielectric properties of ceramics are commonly influenced by extrinsic factors such as the relative density, secondary phases, and microstructure. In the present system, the influence of secondary phases can be considered insignificant, because no impurity phase was detected within the sensitivity of XRD. As shown in Figure 6b–d, the initial increase in Q × f, reaching a maximum at 850 °C, was mainly attributed to continuous microstructural densification and homogenization. The subsequent decrease in Q × f at higher temperatures was associated with the development of microstructural defects and grain-boundary irregularities. Both εᵣ and Q × f changes follow the same trend as the relative density [21], highlighting the dominant role in determining the dielectric response. Additionally, a noticeable improvement in τ_f_ was noted for the sample sintered at 900 °C, suggesting that subtle changes in bond characteristics and local structure at higher temperatures may benefit the temperature stability. This observation provided a useful starting point for future efforts aimed at further optimizing the thermal stability of LCNCZW ceramics. It is commonly accepted that the microwave dielectric properties are governed by both extrinsic and intrinsic factors. The latter include the lattice vibrational modes, ionic polarizability, bond properties, and octahedral distortion. In this work, Raman spectroscopy was employed to investigate the vibrational behavior and intrinsic structural features of the samples [25,26].

**Figure 7:**
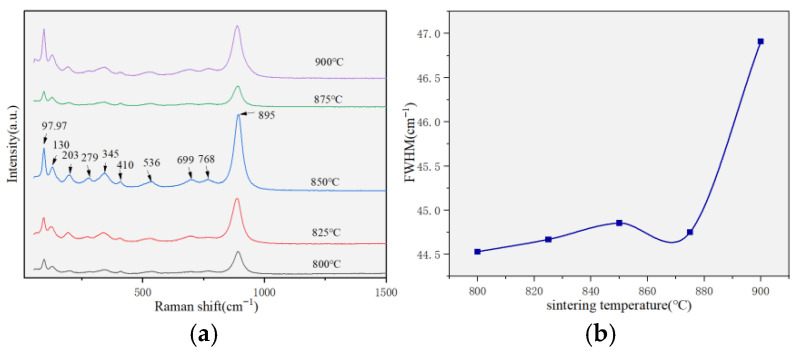
(**a**) Raman spectra of the (Li_0.4_Co_0.2_Ni_0.2_Cu_0.2_Zn_0.2_)WO_4_ high-entropy ceramic; (**b**) FWHM around 895 cm^−1^.

As reported in previous studies [27,28], the Raman vibration modes of wolframite-type ceramics mainly originate from the [ZnO_6_] and [WO_6_] octahedra. In the present LCNCZW ceramics, the strong peak at 895 cm^−1^, shown in Figure 7a, was assigned to the symmetric stretching vibration of W–O bonds. Among all the observed vibrational modes, the [WO_6_] stretching mode near 895 cm^−1^ exhibited the highest frequency and strongest polarity, and therefore made the largest contribution to the microwave dielectric response [26]. Additional Raman modes located at 699, 768, and 410 cm^−1^ are associated with stretching vibrations of longer W–O bonds, while the peaks at 97, 130, 203, and 536 cm^−1^ are related to symmetric stretching vibrations of the [ZnO_6_] octahedra. As shown in Figure 7b, the full width at half maximum (FWHM) of the 895 cm^−1^ peak was obtained through spectral fitting. Only minor changes in FWHM were observed over the investigated sintering temperatures, with values remaining close to 44 cm^−1^. This behavior indicated that the degree of short-range structural disorder is essentially independent of the sintering temperature. The relatively large FWHM values reflected the absence of long-range cation ordering and supported a scenario of random cation distribution over the cationic sublattice, which results in significant structural disorder. Because the FWHM was a sensitive indicator of short-range structural order, it had a strong impact on the τ_f_ value. Although the possible influences of crystallite size and defects cannot be completely excluded, chemical disorder is considered to be the dominant factor.

The lattice energies of individual chemical bonds in the LCNCZW ceramic were evaluated using the P–V–L theory [29,30]. Lattice energy was a useful parameter for predicting and characterizing the physicochemical properties of crystalline materials. Based on the reported structural data of ZnWO_4_ and complex chemical bond theory, the bond-subgroup representation of the (Li_0.4_Co_0.2_Ni_0.2_Cu_0.2_Zn_0.2_)WO_4_ composite crystal can be expressed as

$$\left({Li}_{0.4}{Co}_{0.2}{Ni}_{0.2}{Cu}_{0.2}{Zn}_{0.2}\right){WO}_{4}{=\left({Li}_{0.4}{Co}_{0.2}{Ni}_{0.2}{Cu}_{0.2}{Zn}_{0.2}\right)}_{\frac{1}{3}}{O\left(2\right)}_{\frac{2}{3}}+{\left({Li}_{0.4}{Co}_{0.2}{Ni}_{0.2}{Cu}_{0.2}{Zn}_{0.2}\right)}_{\frac{1}{3}}{O\left(1\right)}_{\frac{2}{3}}^{1}+{\left({Li}_{0.4}{Co}_{0.2}{Ni}_{0.2}{Cu}_{0.2}{Zn}_{0.2}\right)}_{\frac{1}{3}}{O\left(1\right)}_{\frac{2}{3}}^{2}+W_{\frac{1}{3}}{O\left(1\right)}_{\frac{2}{3}}+W_{\frac{1}{3}}{O\left(2\right)}_{\frac{2}{3}}^{1}+W_{\frac{1}{3}}{O\left(2\right)}_{\frac{2}{3}}^{2}$$

Using this formulation, the overall lattice energy of the high-entropy ceramic can be calculated according to Equations (3)–(6):

(3) $$U_{cal}=\sum_{\mu }U_{b}^{\mu }$$

(4) $$U_{b}=U_{bc}^{\mu }+U_{bi}^{\mu }$$

(5) $$U_{bc}^{\mu }=2100m\frac{{\left(Z_{+}^{\mu }\right)}^{1.64}}{{\left(d^{\mu }\right)}^{0.75}}f_{C}^{\mu }$$

(6) $$U_{bi}^{\mu }=1270\frac{\left(m+n\right)Z_{+}^{\mu }Z_{-}^{\mu }}{d^{\mu }}\left(1-\frac{0.4}{d^{\mu }}\right)f_{i}^{\mu }$$

where $U_{bc}^{\mu }$ and $U_{bi}^{\mu }$ represent the covalent lattice energy and ionic lattice energy, respectively; $f_{C}^{\mu }$ and $f_{i}^{\mu }$ [31] denote the covalent factor and ionic factor; $Z_{+}^{\mu }$ and $Z_{-}^{\mu }$ are the valences of the cation and anion in the μ-type chemical bond; *d_μ_* is the bond length; and m and n indicate the numbers of cations and anions involved in the μ-type bond.

The total lattice energy corresponds to the sum of all interionic interaction potentials. Higher U_total_ values are indicative of greater thermodynamic stability and a more robust crystal structure, and are associated with a corresponding reduction in losses caused by anharmonic vibrations.

**Figure 8:**
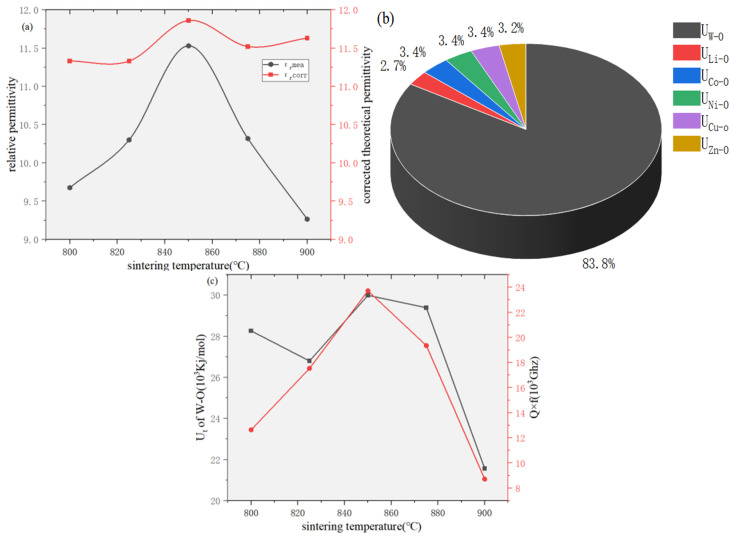
(**a**) variation in the measured relative permittivity (εᵣ) and theoretical permittivity (ε_rc_); **(b**) bond energy; (**c**) bond energy of the W–O bond (U_w–o_) plotted against the Q × f value.

**Figure 9:**
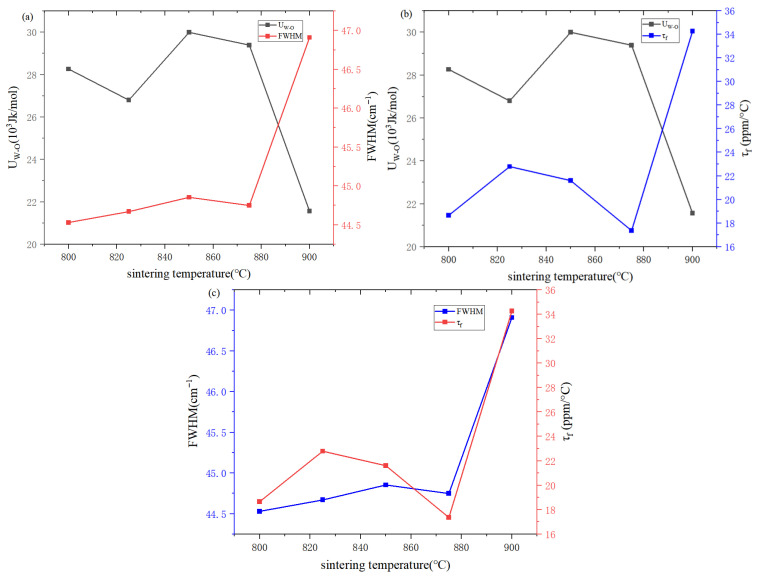
(**a**) U_w-o_ versus and FWHM variation; (**b**) U_w-o_ versus and τ_f_ variation; (**c**) FWHM versus and τ_f_ variation.

The intrinsic relationship between ε_r_ and $f_{i}$ can be estimated by Equation (7) [32]:

(7) $$\varepsilon_{r}=\frac{n^{2}-1}{1-f_{i}}+1$$

where n represents the refractive index. In the calculation of the theoretical permittivity, we only considered the refractive index of conventional single-phase ZnWO_4_, because the PDF card of the high-entropy ceramic (Li_0.4_Co_0.2_Ni_0.2_Cu_0.2_Zn_0.2_)WO_4_ corresponds to that of single-phase ZnWO_4_ and exhibits the same crystal structure. The calculated theoretical permittivity is slightly higher than the experimental value, which can be attributed to limitations of the theoretical model, experimental measurement errors, porosity, and other factors. The W–O bond in Figure 8a is shown to exhibit moderate bond ionicity, while tungsten (W) possesses the highest polarizability among the ions (ionic polarizabilities: Li^+^ 1.20 Å^3^, Co^2+^ 1.65 Å^3^, Ni^2+^ 1.23 Å^3^, Cu^2+^ 2.11 Å^3^, Zn^2+^ 2.04 Å^3^, W^6+^ 3.20 Å^3^ [33,34], O^2−^ 2.01 Å^3^). This is indicative of the significant influence of the W–O bond on ε_r_. Nevertheless, the bonds between the A-site cations and oxygen also play an important role. For example, as shown in Table 2, the ionicity (fᵢ) and lattice energy (U) of the Co–O, Ni–O, Cu–O, and Zn–O bonds exhibit non-monotonic variations with sintering temperature, which correlate with the fluctuations in lattice parameters. Although these variations are less dramatic than those of the W–O bond, they collectively influence the overall lattice vibrational characteristics and dielectric loss. Lattice energy, which represents the strength of cation–anion bonding, is closely linked to crystalline stability. As shown in Figure 8b, the W–O bond is characterized by a notably high lattice energy (U), implying its significant contribution to the structural integrity of the lattice and its positive role in enhancing the Q × f value [35]. The temperature stability of the material is assessed using the τ_f_ parameter, with values closer to zero indicating superior stability. This parameter is predominantly determined by the degree of structural ordering and the stability of chemical bonds, which correlate respectively with the Raman FWHM and bond energy. The variation in τ_f_, as revealed in Figure 9, follows a similar trend to the total bond energy of the W–O bond, supporting the view that greater bond energy enhances bond stability and thereby improves thermal performance [35]. Notably, at a sintering temperature of 900 °C, the concurrent reduction in W–O bond energy and increase in FWHM lead to diminished structural ordering and a deterioration in temperature stability.

To facilitate a direct comparison between the high-entropy ceramic in this study and conventional ZnWO_4_, we summarize the relevant properties in Table 3. Compared with pure ZnWO_4_ reported in reference, the (Li_0.4_Co_0.2_Ni_0.2_Cu_0.2_Zn_0.2_)WO_4_ ceramic exhibits a significantly lower sintering temperature (850 °C vs. >1100 °C), demonstrating the remarkable advantage of the high-entropy design in improving sinterability—a critical factor for LTCC applications. Although the Q × f value obtained in this work (~23,851 GHz) is lower than that of pure ZnWO_4_ (~60,000 GHz), which may be attributed to enhanced phonon scattering due to lattice distortion in the high-entropy system, both εᵣ and τ_f_ are substantially improved. Notably, τ_f_ is adjusted from ~−60 ppm/°C to a near-zero value of +21.6 ppm/°C, illustrating the great potential of the high-entropy strategy in tuning thermal stability. Thus, despite its limitations in achieving ultra-low loss, this material shows unique advantages in applications that require moderate Q × f, adjustable τ_f_, and low-temperature sintering.

## 4. Conclusions

The (Li_0.4_Co_0.2_Ni_0.2_Cu_0.2_Zn_0.2_)WO_4_ high-entropy ceramics were successfully synthesized via a conventional solid-state reaction method at 800–900 °C. All samples crystallized into a single-phase structure with the space group P2/c, confirming the complete incorporation of Li^+^, Co^2+^, Ni^2+^, Cu^2+^, and Zn^2+^ into the tungstate lattice. Based on complex bond valence theory, the chemical bond properties were analyzed, revealing the critical role of the W–O bond in governing the dielectric response. The increase in Q × f observed at 850 °C is attributed to progressive microstructural densification and lattice compression, which enhance the W–O bond energy and improve structural stability. At higher sintering temperatures (875 and 900 °C), the reduction in Q × f is mainly associated with weakened structural stability. In addition, the thermal stability of the ceramics is influenced by the degree of structural ordering and the stability of the chemical bond, which are closely related to the Raman FWHM and W-O bond energy, respectively. The optimal microwave dielectric properties (εᵣ = 11.697 ± 0.204, Q × f = 23,851 ± 0.126 GHz, and τ_f_ = 21.335 ± 0.232 ppm/°C) were achieved in the sample sintered at 850 °C, indicating that the high-entropy design effectively improves sinterability and reduces dielectric loss in wolframite-structured tungstate ceramics. Therefore, the (Li_0.4_Co_0.2_Ni_0.2_Cu_0.2_Zn_0.2_)WO_4_ high-entropy ceramic is a promising candidate for applications in microwave devices.

## Figures and Tables

**Table 1:** Refinement data for LCNCZW ceramics sintered at different temperatures.

	800 ^(a)^	825 ^(b)^	850 ^(c)^	875 ^(d)^	900 ^(e)^
R_wp_ (%)	7.856	9.145	9.145	8.731	7.856
a (Å)	4.647	4.653	4.649	4.653	4.652
b (Å)	5.698	5.707	5.699	5.707	5.702
c (Å)	4.916	4.921	4.918	4.922	4.924
GOF	2.27	2.58	2.27	2.38	2.02
V_m_ (Å^3^)	130.193	130.721	130.339	130.710	130.656

^(a)^ sinting temperature 800 °C; ^(b)^ sinting temperature 825 °C; ^(c)^ sinting temperature 850 °C; ^(d)^ sinting temperature 875 °C; ^(e)^ sinting temperature 900 °C.

**Table 2:** Lattice energies of individual chemical bonds in the LCNCZW ceramic.

Chemical Bond	*U* (kJ/mol)						
	$f_{i}^{\mu }$	$f_{C}^{\mu }$	800 °C	825 °C	850 °C	875 °C	900 °C
Li1-O(1)	0.6020	0.3979	731.39	517.56	489.36	428.04	489.24
Co1-O(1)	0.3782	0.6217	774.36	642.11	583.30	531.05	606.77
Ni1-O(1)	0.3691	0.6308	575.52	647.23	587.16	535.26	611.60
Cu1-O(1)	0.3722	0.6277	773.60	645.54	585.90	543.43	610.01
Zn1-O(1)	0.4447	0.5552	760.40	605.39	555.55	500.62	572.03
Li2-O(2)	0.6020	0.3979	467.39	489.20	510.35	530.81	474.42
Co2-O(2)	0.3782	0.6217	576.63	606.70	633.09	658.75	588.36
Ni2-O(2)	0.3691	0.6308	584.24	611.53	638.14	672.00	593.03
Cu2-O(2)	0.3722	0.6277	1582.72	609.94	636.48	662.27	591.49
Zn2-O(2)	0.4447	0.5552	546.66	571.95	596.79	620.90	554.65
W1-O(1)	0.4374	0.5626	28,263.13	26,799.85	29,990.83	29,391.03	21,588.13

**Table 3:** Comparison of properties between conventional ZnWO_4_ and high-entropy (Li_0.4_Co_0.2_Ni_0.2_Cu_0.2_Zn_0.2_)WO_4_.

Material	Sintering Temp. (°C)	ε_r_	Q × f (GHz)	τf (ppm/°C)	Reference
ZnWO_4_	>1100	~15	~60,000	~−60	[14]
(Li_0.4_Co_0.2_Ni_0.2_Cu_0.2_Zn_0.2_)WO_4_	850	11.529	23,714	21.6	This work

## Data Availability

The original contributions presented in this study are included in the article. Further inquiries can be directed to the corresponding author.
